# Fc-Engineered Therapeutic Antibodies: Recent Advances and Future Directions

**DOI:** 10.3390/pharmaceutics15102402

**Published:** 2023-09-28

**Authors:** Dalia T. Abdeldaim, Katharina Schindowski

**Affiliations:** 1 Institute of Applied Biotechnology, University of Applied Science Biberach, 88400 Biberach, Germany; abdeldaim@hochschule-bc.de; 2 Graduate School for Cellular and Biomedical Sciences, University of Bern, 3012 Bern, Switzerland

**Keywords:** tailored antibodies, cancer therapy, half-life-extended antibodies, Fc engineering, protein engineering, bispecific antibodies

## Abstract

Monoclonal therapeutic antibodies have revolutionized the treatment of cancer and other diseases. Fc engineering aims to enhance the effector functions or half-life of therapeutic antibodies by modifying their Fc regions. Recent advances in the Fc engineering of modern therapeutic antibodies can be considered the next generation of antibody therapy. Various strategies are employed, including altering glycosylation patterns via glycoengineering and introducing mutations to the Fc region, thereby enhancing Fc receptor or complement interactions. Further, Fc engineering strategies enable the generation of bispecific IgG-based heterodimeric antibodies. As Fc engineering techniques continue to evolve, an expanding portfolio of Fc-engineered antibodies is advancing through clinical development, with several already approved for medical use. Despite the plethora of Fc-based mutations that have been analyzed in in vitro and in vivo models, we focus here in this review on the relevant Fc engineering strategies of approved therapeutic antibodies to finetune effector functions, to modify half-life and to stabilize asymmetric bispecific IgGs.

## 1. Antibodies—Magic Bullets in Therapy

Antibodies are proteins that are produced by B cells and are an important part of the humoral defensive immune system to attack any kind of foreign structure. Despite their susceptibility to off-target binding, they are considered very specific, meaning that each antibody usually binds to one particular antigen. This specificity makes antibodies ideal for use in therapy, as they can be targeted to specific cells, such as cancer cells [1]. The concept of the medical term “Zauberkugel” (German for Magic Bullet) was initially conceived by Paul Ehrlich, a German scientist, who, together with the Russian scientist Ilya Metchnikov, received the Nobel Prize in Physiology and Medicine in 1908 [2,3]. A Magic Bullet is a drug that targets and destroys specific disease-causing toxins, microbes or cells without harming the patient’s own organism. Based on Ehrlich’s early research on serum-based antibodies, these proteins are the closest that we have to a Magic Bullet: clinical antibodies are used to target and destroy a wide range of diseases, including cancer, infections and autoimmune disorders.

Endogenous antibodies are produced in response to the exposure of a foreign structure, such as microbes, parasites or cancer antigens. Although these immunoglobulins (Igs) produced in humans have different classes, such as IgA, IgD, IgE, IgG and IgM, the therapeutic interest is currently mainly based on the “Y”-shaped IgG proteins [4].

### 1.1. Molecular Structure of Immunoglobulin G (IgG)

**Figure 1:**
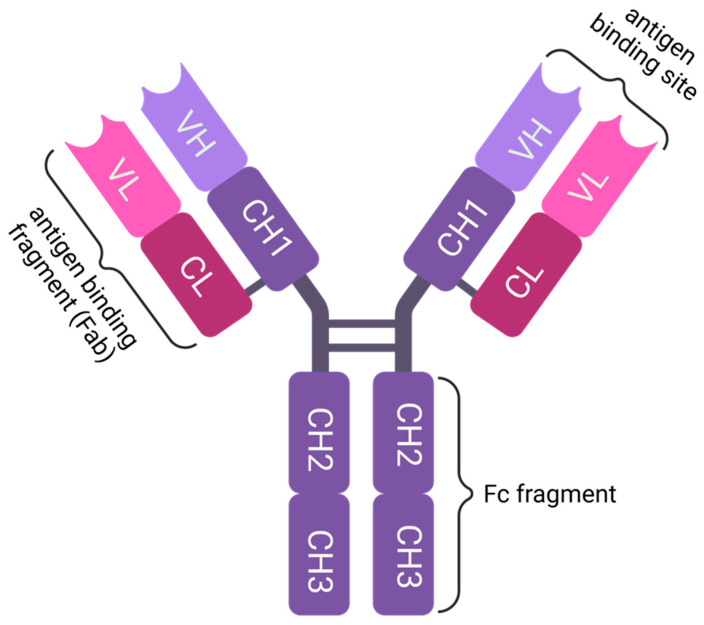
IgG is a Y-shaped protein made up of four polypeptide chains: two identical heavy chains and two identical light chains. The heavy chains are about 50 kDa in size, while the light chains are about 25 kDa. The chains are linked together by disulfide bonds, which form between cysteine residues on the respective chains. The antigen-binding site is located at the tips of the Y-shaped structure, where the variable domains of the light and heavy chains form the antigen-binding variable fragment. The Fc region or Fc fragment is located at the base of the Y-shaped structure and is usually N-glycosylated at N297. The Fc region is responsible for the effector functions of IgG, e.g., complement activation (CDC), antibody-dependent cellular phagocytosis (ADCP) and antibody-dependent cellular cytotoxicity (ADCC). Created with BioRender.com.

A human IgG consists of two identical glycosylated heavy chains, each approximately 50 kDa, and two identical light chains, each approximately 25 kDa [4,5]. Both the heavy and light chains have variable (N-terminal) and constant regions (C-terminal) and are linked to each other by disulfide bonds (see Figure 1). The heavy chain consists of one variable region (VH) and three constant regions (CH1, CH2 and CH3). In contrast, the light chain consists of only one variable region (VL) and one constant region (CL) [6]. The entire light chain (VL and CL) and the upper part (VH and CH1) of the heavy chain constitute the Fab fragment. The remainder of the heavy chain (CH2 and CH3) forms the Fc region of the IgG. The Fab fragment and the Fc region are linked together by the so-called hinge region [6]. In the case of the IgG antibody class, the Fc region is referred to as Fc.

**Figure 2:**
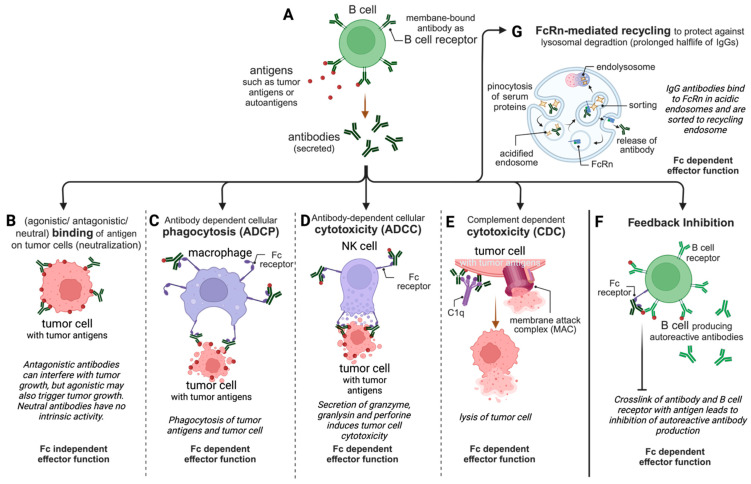
Illustration of the relevant effector functions of approved therapeutic antibodies. (**A**) IVIg or hyperimmune sera are produced from B cells of healthy donors or convalescent patients. (**B**) Neutralization is probably the best-known activity of therapeutic antibodies and is responsible for highly specific binding to the antigen with high affinity. (**C**) ADCP, where antibodies bind to target cells or molecular structures and make them more susceptible to phagocytosis, is mostly used for tumor targeting. This effector function is mediated by several FcγRs. (**D**) ADCC is mediated by FcγRIIIA: therapeutic IgGs bind to target cells and recruit natural killer (NK) cells to kill the target cells. Also, this effector function is highly relevant for cancer therapy. (**E**) CDC, where antibodies bind to target cells, activate the complement via C1q and finally lyse target cells via the MAC, is of relevance to cancer therapy. (**F**) FcγRIIB is the only known inhibitory FcR and inhibits innate and adaptive immunity via several pathways. Targeting the feedback inhibition of B cells is not only relevant for the therapy of allergies but also for improved tumor targeting. (**G**). The FcRn-mediated recycling of proteins that contain an Fc region is responsible for the long plasma half-lives of IgGs. See the main text for details. Created with BioRender.com.

While the only known function of the antigen-binding site is to bind the antigen, which is, in several cases, equivalent to neutralizing the antigen, the constant Fc region of an antigen–antibody complex triggers several humoral functions [7]. Via the binding of the Fc region to the Fc receptors on immune cells or to the complement, antibodies are able to initiate several effector functions that lead to the killing of microbes or of virus-infected cells or tumor cells [2]. As indicated in Figure 2, IgG’s effector functions are not only used in tumor therapy but also in immunotherapy and several more therapeutic areas.

### 1.2. Therapeutic Antibodies—How It All Started

The history of monoclonal antibodies is a long and fascinating one, dating back to the early days of immunology. In 1890, Emil von Behring and Shibasaburo Kitasato discovered that serum from animals immunized against diphtheria could protect other animals from the disease. This discovery led to the development of the first antibody-based therapy, diphtheria antitoxin [8]. In the early 1900s, Paul Ehrlich’s Magic Bullets described the ideal antibody-based therapy, which would be highly specific and would only target the disease-causing organisms [3]. In the 1940s, the first human monoclonal antibodies (mAbs) were produced, but they were not used in clinical therapy due to their high cost and difficulty in production [3]. The development of hybridoma technology by Milstein and Köhler in 1975 enabled the production of larger quantities of monoclonal antibodies at a relatively low cost [2,3,9]. This led to the development of the first monoclonal antibody-based therapy, muromonab-CD3 (Orthoclone OKT3), which was used to treat acute rejection of organ transplants [10]. At that time, predominantly murine antibodies had been generated using hybridoma technology, including muronomab-OKT3, which is a murine IgG that was administered to humans. But, the primary drawback of murine monoclonal antibodies lies in their limited tolerance in human recipients, posing a risk of immunogenicity among patients. This can lead to the production of human anti-mouse antibodies (HAMAs), which can interfere with the therapeutic effects of murine mAbs and can also cause side effects, such as a loss of efficacy and allergic reactions [4]. In the 1980s, Rituximab (Rituxan) was approved for the treatment of non-Hodgkin’s lymphoma, thereby becoming the first antibody for the treatment of cancer [11]. Rituximab is a chimeric mAb that reduces the risk of immunogenicity associated with murine mAbs [12]. Chimeric mAbs are developed via genetic engineering, where only the murine antigen-specific variable region is kept, and the remaining light and heavy chains are derived from human antibodies, resulting in mAbs that are 65% human and 35% murine and, thus, better tolerated [12,13]. Trastuzumab (Herceptin) was approved for the treatment of HER2^+^ breast cancer in the 1990s, becoming the first mAb for the treatment of solid tumors [14]. Humanized mAbs such as Trastuzumab are generated by engineering the murine hypervariable regions of heavy and light chains onto a human antibody framework to be even better tolerated by the human immune system [13]. In the 2000s, Adalimumab (Humira) was approved for the treatment of rheumatoid arthritis, a chronic autoimmune disease [15]. Adalimumab is a fully human antibody and the first mAb approved that derived from phage display [16]. Human antibodies can also be generated in transgenic animals like mice carrying human Ig genes whose endogenous Ig genes have been silenced [17]. In the 2010s, not only was Omalizumab (Xolair) approved for the treatment of allergic asthma, but also the first Fc- and glyco-engineered antibodies were approved [18]. Now, in the 2020s, monoclonal antibodies are used to treat a wide range of diseases, including cancer, autoimmune diseases and infectious diseases. For additional details about the antibodies mentioned in this review, refer to the Supplementary Data in Table S1.

## 2. IgG Effector Functions—The Gunpowder of Magic Bullets

The basic function of an Ig being expressed with a transmembrane domain is to serve as a B-cell receptor (Figure 2A) [4]. Upon activation, some B cells start to differentiate into plasma cells and splice off the transmembrane region of the B-cell receptor’s mRNA, resulting in a secretory protein—an antibody. Polyclonal hyperimmune antibody products are derived from plasma donors who have a proven immunity against specific infectious diseases, such as viral hepatitis, chickenpox, rabies and COVID-19 [4]. These plasma-derived antibody products have shown promising results in viral diseases like H5Z1 influenza and Ebola viral disease and have improved severe cases of COVID-19 [19]. In addition, purified polyclonal IgGs from a pool of healthy donors are used in the therapy of autoimmune disease or for patients with immune suppression as intravenous immunoglobulin G (IVIg). Monoclonal therapeutic IgGs are mostly produced in expression systems such as Chinese hamster ovarian (CHO) cells and share the activities detailed below with polyclonal products, which are relevant for their clinical efficacy.

Apart from neutralization, all other clinically relevant IgG effector functions are dependent on the presence of an Fc region that interacts with either an Fcγ receptor (FcγR) on the surface of a cell or the complement component 1q (C1q). Further, IgGs interact with the neonatal Fc receptor (FcRn). The cellular immune response is mediated by either four activating receptors (FcγRI, FcγRIIA, FcγRIIC and FcγRIIIA) through their intracellular immunoreceptor tyrosine-based activation motif (ITAM) or an inhibitory receptor (FcγRIIB) that signals through an immunoreceptor tyrosine-based inhibitory motif (ITIM) [7,20,21]. Interestingly, FcγRIIIB is connected to neither an ITAM nor an ITIM, and, hence, it is believed to serve as an IgG trap [22].

### 2.1. Fc-Independent Binding/Neutralization

The neutralization or blocking of an antigen is the only activity of antibodies that is independent of the Fc region (Figure 2B); hence, antibody fragments, single-chain variable fragments (scFvs) and single-domain antibodies (sdAbs) can also effectively neutralize antigens, e.g., Abciximab, Ranibizumab, Certolizumab Pegol, Idarucizumab, Brolucizumab and Caplacizumab [23]. In contrast to this, antibodies can also act as agonists in case the antigen is a molecule with intrinsic activity such as a receptor [24]. The anti-CD28 superagonistic TGN1412 is an example of the catastrophic clinical consequences of an unexpected superagonism [25].

The neutralization of the antigen is undoubtedly of high importance in the fight against infectious disease, but we learned from the SARS-CoV-2 pandemic that even non-neutralizing antibodies, which were observed when the virus evolved, are highly affected by Fc-mediated effector functions [7]. Likewise, some therapies focus on the neutralization of mediator molecules (such as tumor necrosis factor-alpha (TNF), vascular endothelial growth factor (VEGF) and different interleukins (ILs)) or pathogens (such as *B. anthracis*), but it is well documented that Fc-dependent effector functions also have some advantages in the disease control of rheumatoid arthritis or in anthrax animal models [26].

### 2.2. Fc-Dependent Antibody-Dependent Cell-Mediated Phagocytosis (ADCP)

The specific binding of the IgG to an antigen and the formation of immune complexes is also referred to as opsonization, as this increases the visibility of the antigen to phagocytes. These antigen–antibody complexes can then be captured by several FcγRs on the surfaces of macrophages, monocytes, dendritic cells or neutrophils and activate these phagocytes to induce antibody-dependent cell-mediated phagocytosis (ADCP, Figure 2C) [27]. This includes the internalization of the immune complexes binding to FcγRs, trafficking to lysosomes and the phagolysosomal degradation of the antigens to peptides. These antigen-derived peptides are then loaded onto MHC molecules for presentation to T cells [4,7]. Human macrophages express the activating receptors FcγRI, FcγRIIA and FcγRIIIA, as well as inhibitory FcγRIIB [4]. The involvement of ITAM- and ITIM-bearing FcγRs in ADCP has been reported, but ITIM signaling appears to be involved in the retention of the complexes for subsequent transfer to B cells [26,28]. The role of ADCP in the depletion of B-cell lymphoma cells was demonstrated in clinical studies with Rituximab, linking the FcγRIIA-131H/R polymorphism with the clinical outcome of the mAb therapy [29,30]. Also, in infectious disease such as COVID-19, SARS-CoV-2-specific antibodies mediating ADCP were associated with survival in hospitalized patients [31]. Interestingly, the same study reported that virus-specific antibodies, which signal and mediate ADCP through FcγRIIIB in neutrophils, were associated with more severe disease [7,31].

### 2.3. Fc-Dependent Antibody-Dependent Cell-Mediated Cytotoxicity (ADCC)

IgGs bound to antigens on the surface of cells (such as tumor antigens on cancer cells) crosslink FcγRIIIA (also known as CD16A) on natural killer (NK) cells and activate through their ITAM antibody-dependent cell-mediated cytotoxicity (ADCC, Figure 2D) [32]. During ADCC, NK cells form an immune synapse with the target cell and secrete perforin and granzymes that induce the apoptosis and lysis of the target cell [20]. Crosslinking with IgG-containing complexes involves an interaction between the Fc hinge region/CH2 and FcγRIIIA and is dependent on the glycan present at the conserved *N*-glycosylation site Asparagine 297 (N297) in each of the CH2 domains [33,34]. However, the crosslinking of the receptors by IgG–antigen complexes is pivotal since monomeric IgG interactions with low-affinity FcγRIII results in inhibition by increasing the thresholds of activation; this is called inhibitory ITAM (ITAMi) signaling [35]. In humans, two different alleles of FcγRIIIA encode a variant with either valine (V158) or phenylalanine (F158) at position 158. The FcγRIIIA-V158 variant is reported to have a 10-fold higher affinity to human IgG1 than FcγRIIIA-F158 [36].

ADCC has been evaluated extensively in mAb-based tumor therapy, e.g., Rituximab, Herceptin or Cetuximab [37]. Additionally, the Fc regions’ glycan forms have been intensively studied and exploited to improve ADCC (see also Section 3.2).

### 2.4. Fc-Dependent Complement-Dependent Cytotoxicity (CDC)

Complement components are found ubiquitously in the mammalian blood and tissue. IgGs, except for IgG4, can recruit complement after they bind to cell-surface-bound antigens (Figure 2E) [4,38]. In particular, stable hexameric IgG–antigen complexes are optimal for recruitment since C1q itself forms a hexamer.

The complement cascade can be activated via three distinct pathways, and two out of these three are involved in the IgG effector function complement-dependent cytotoxicity (CDC) [26]. The recruitment of C1q to the immune complex is referred to as the classical pathway. This event is followed by the association of C1q with other complement components of the C1 complex and the recruitment of C3 on the surface of the antigen-bearing target cell. From here, the alternative pathway starts, which ends with the assembly of the membrane attack complex (MAC), a membrane pore that directly lyses the target cell [39,40]. The IgG1 interaction site for C1q recruitment is located in the lower hinge region and the upper part of the CH2 domain [38].

### 2.5. Fc-Dependent Inhibitory Effects

FcγRIIB, the only inhibitory Fc receptor, functions to suppress the hyper-activation of immune cells through its ITIM [41]. Therefore, it is known to control humoral immunity by regulating B-cell activation and plasma cell survival to inhibit (1) the antigen presentation of dendritic cells to T cells, (2) dendritic cell maturation, (3) FcγR-mediated phagocytosis and cytokine release in macrophages and, last but not least, (4) IgE-induced mast cell and basophil degranulation [42]. Hence, inhibition through FcγRIIB is critical for balanced immunity, and a single-nucleotide polymorphism leading to I232T substitution within the transmembrane domain of FcγRIIB is associated with an increased risk of autoimmune diseases [41].

The inhibiting effect of FcγRIIB on B cells, the so-called feedback inhibition (Figure 2F), is initiated by the binding of IgGs to B-cell-expressed FcγRIIB and crosslinking with the B-cell receptor via a shared antigen. While, usually, B cells are activated upon the binding of the B-cell receptor to the antigen, here, FcγRIIB inhibits B-cell activation through ITIM [42]. Feedback inhibition can be used in the therapy of allergies and allergic asthma to decrease the titer of anti-allergenic IgE (see Section 3.5.3).

In tumor therapy, however, any FcγRIIB-mediated inhibition of cytotoxic effector functions is undesired and may be related to poor clinical outcomes [43,44]. Hence, Fc engineering decreasing the affinity of IgGs to FcγRIIB is discussed to improve cancer therapy.

### 2.6. Fc-Dependent FcRn-Mediated Transport of IgGs Resulting in a Prolonged Serum Half-Life and Mucosal Immunity

The neonatal Fc receptor (FcRn) was named as such because of the observation that passive short-term humoral immunity is transferred from the mother to the fetus in utero, as well as via breast milk to newborns [45]. In adults, FcRn is expressed on several cells and tissues, e.g., endothelial cells, monocytes, mucosa and muscles.

The systemic clearance, that is, the half-life, of proteins is mostly determined by non-metabolic elimination pathways like renal clearance, unspecific metabolic pathways like proteolysis inside the cells following pinocytosis and specific metabolism pathways that involve receptor-mediated endocytosis and degradation [46]. It is mainly the size of the proteins, as well as their charge state and glycosylation pattern, that is relevant for renal clearance. While most serum proteins with a size above the threshold of renal clearance still have a rather shorter plasma half-life of several hours up to a few days, IgGs and serum albumin exhibit in humans a half-life of one week (IgG3) up to three weeks (albumin, IgG1, IgG2 and IgG4) [4]. Serum proteins are passively pinocytosed by endothelial cells and monocytes into acidified endosomes (Figure 2G). Via fusion with endosomes containing FcRn, all proteins with an Fc region, as well as albumin, may bind to FcRn at low pH. A sorting process directs all unbound proteins to lysosomal degradation, whereas IgG, albumin or Fc fusion proteins bound to FcRn are either recycled to the cellular surface and released back into the serum at physiologic pH or are transcytosed. The FcRn-mediated transcytosis of IgGs, in combination with the polymeric Ig receptor-mediated transcytosis of IgA and IgM, plays an important role in mucosal immunity [20]. FcRn-IgG interaction takes place at the junction of the CH2 and CH3 domains in a pH-dependent manner. Optimal binding occurs at low pH (pH < 6.5) in acidified endosomes, while binding at pH > 6.5 is poor; hence, physiological pH results in the dissociation of the FcRn-IgG complex. The pH dependence is regulated by the protonation of H310, H435 and H436 in the Fc region at low pH; therefore, these positively charged residues bind to negatively charged residues within the FcRn [47,48]. In the human IgG3 subtype, His435 is replaced with R435 with a pKa of 12.5. Thus, the half-life of IgG3 is reduced to 7 days instead of 21 days for IgG1, IgG2 and IgG4 [47,48].

This highly efficient recycling mechanism via FcRn is responsible for IgG and albumin making up to 90% of the serum protein content, and it was estimated that the FcRn-mediated IgG recycling rate is 42% greater than the rate of IgG production [49]. Thus, inhibiting FcRn recycling can be used in therapy to wash out autoreactive antibodies in autoimmune diseases [45].

## 3. Specific Fc-Based Mutations and Fc Glycoengineering to Improve Clinical Outcomes

The molecular understanding of Fc–FcγR interactions results in an increasing number of Fc-engineered therapeutic antibodies with activities tailored for specific applications. Also, Fc glycosylation plays a central role in antibody function, and glycoengineering has the potential to tailor the effector function of therapeutic antibodies.

### 3.1. The Impact of Glycosylation and Aglycosylation

Glycoengineering, used to remove the fucose from an IgG1 glycan, is the oldest among all clinically established Fc engineering techniques, with Obinutuzumab being approved in 2013 [26]. Nevertheless, the impact of the glycan structure on effector function is far from being elucidated. The quaternary structure of IgG1 is stabilized by N-linked glycosylation, and it also increases the solubility of the Ig. Although the IgG1 Fc region contains only one glycosylation site at asparagine 297 (N297), at least 36 different glycan structures can be attached, and each glycoform has a different impact on FcγR and probably also on FcRn interaction [50]. The glycosylation of other subtypes is even more complex due to there being more glycosylation sites, as well as O-linked glycosylation. Interestingly, as both heavy chains harbor glycosylation sites, glycan heterogeneity may occur within one IgG molecule [51].

A typical IgG1 glycan is a biantennary glycan consisting of two N-acetylglucosamine (GlcNAc) glycans, three mannose glycans, and two more GlcNAc glycans linked to the mannose [51]. Fucose, galactose, sialic acid and GlcNAc can also be added to this glycan core.

Completely removing the glycan, either via enzymatic cleavage or by mutating the glycosylation site, results in the elimination of Fc effector functions [52,53]. The effects are reported to range from a markedly reduced binding of aglycosylated IgG1 to FcγRI and C1q up to a complete loss of FcγR binding and, therefore, the ability to initiate ADCC for aglycosylated IgG3 [54]. It also appears that the stability of an aglycosylated IgG1 and IgG4 is impaired in particular in the CH2 domains, resulting in a closed conformation of the Fc region [55,56], while an open conformation is needed for FcγR binding [57]. Moreover, proteolytic degradation is more likely to occur when an IgG is glycosylated [53,58]. However, the half-life of aglycosylated IgGs is mostly not affected [59].

The loss of effector functions is an advantage for therapeutic antibodies inhibiting the so-called immune checkpoints in cancer (see also Section 3.5.2). Atezolizumab (Tecentriq) is a human IgG1 mutated at N297A which results in aglycosylation, and was approved in 2016 for several cancers. It not only targets programmed cell death 1 ligand 1 (PD-L1) but also displays reduced stability and tends to form aggregates [60].

### 3.2. Reduced Fucosylation and Afucosylation for Improved ADCC

An in-depth IgG glycome study of different human populations demonstrated that the majority of endogenous serum IgGs are fucosylated with a fucose at the core biantennary glycan structure [61]. Afucosylated IgGs levels were determined from 1.3% to 19.3% and explained the different efficacies observed in the ADCC of endogenous IgGs. During viral infections, the levels of core fucosylation decreased, which was associated with improved antiviral activity.

Interestingly, the removal of this core fucose from the biantennary structure of the IgG1 glycan enhances FcγRIIIA interaction and therefore ADCC [26]. The fucose of the core-fucosylated biantennary structure was reported to interfere with the glycan of FcγRIII, thereby sterically hindering tight binding [62]. Obviously, it is not only the size of the fucose causing the hindrance since even bulkier glycan structures such as G2F improve FcγRIII binding when they stabilize the so-called horseshoe conformation of the two CH2 domains. This notion can be particularly exploited in the context of recombinant therapeutic antibody production.

GDP-fucose is enzymatically transferred to the glycan by several different fucosyltransferases (FUTs), depending on the glycan structure. It is important to note that, during recombinant IgG production, the glycan composition may be altered due to the endogenous FUT levels of the expression system [63]. As CHO cells are the most prominent expression system for IgG, it is worth having a closer look at the enzymatic machinery responsible for glycan formation. The overexpression of N-acetylglucosaminyltransferase III (GnTIII) results in reduced core fucosylation [64], as well as the attachment of a bisecting GlcNAc residue [65]. Combined with the expression of α-mannosidase II, this GnTIII-overexpressing cell line named GlycoMab technology was exploited by Glycart in 1999, which was the start of cell-line-driven glycoengineering [44]. In 2013, Obinutuzumab was the first approved antibody produced with Glycart’s GlycoMab technology. Obinutuzumab is approved to treat lymphoma. Another option is to knock out FUT to decrease core fucosylation. This approach was demonstrated by knocking out FUT8 in CHO cells to produce afucosylated antibodies and exploited using POTELLIGENT technology [66]. Mogamulizumab was the first approved afucosylated antibody for the treatment of lymphoma [58]. A comparison of the therapeutic chimeric IgGs produced in CHO, NS0 and rat hybridoma YB2/0 cells revealed the highest ADCC activity in the product produced by YB2/0 [67]. The only difference between these antibodies was the presence of the bisecting N-acetylglucosamine glycan in the YB2/0 product. Thus, Ublituximab (Briumvi) produced in YB2/0 cells was approved for the treatment of multiple sclerosis in 2022 [67].

Other sugar molecules also have an impact on IgG effector activity. Fc galactosylation enhances FcγRIIIA and C1q binding [68,69]. Although, for ADCC, this was shown to be of a much lesser significance than removing the fucose core from the Fc portion of IgG, the effect on CDC was well pronounced [70,71].

### 3.3. Improving ADCC with the Introduction of Mutations

Resolving the molecular structures of Fc-FcγR interactions encouraged the engineering of the Fc region to modulate effector function. A pioneer study in this context was an extensive alanine scanning mutagenesis of the surface amino acid residues and characterization of the mutants in regard to FcγRs, as well as FcRn binding [72]. This study paved the way for many approaches of Fc engineering and for approved therapeutical Fc-engineered antibodies. S239 and I332 were identified to be relevant for FcγRIIIA binding, and several preclinical studies demonstrated that the mutations S239D/I332E increased the binding affinity to FcγRIIIA and FcγRIIB, which translated to enhanced ADCC without altering CDC [73]. Tafasitamab (Minjuvi/Monjuvi) is an anti-CD19 human IgG1/2 chimera harboring the mutations S239D/I332E. It was approved in 2020 for the therapy of lymphoma and is based on XMab technology [74]. BI 836826, an anti-CD37 chimeric IgG1 currently in a phase 2 trial against relapsed/refractory chronic lymphocytic leukemia, also contains S239D/I332E [44,75]. Likewise, A330 was one of the identified amino acids important for FcγR binding, and, in combination with two to three other relevant mutations, the mutation A330L contributed to enhanced affinity to FcγRIIA, FcγRIIB and FcγRIIIA in vitro and a higher removal of target cells in vivo [76,77]. However, it seems that not all clinical indications benefit from as many ITAM-linked effector functions as possible: The basic principle of checkpoint inhibition is to exclusively block the interaction and therefore the unwanted signaling between the two cells forming the immune synapse. Nevertheless, the elimination of regulatory T cells in the tumor environment was discussed as a potential benefit of the therapy [78]. Thus, Zalifrelimab, a checkpoint inhibitor targeting CTL-4, was Fc-engineered at positions S239D/A330L/I332E [44]. Surprisingly, it seems that the clinical outcome is comparable to that of a non-Fc-engineered anti-CTLA-4 mAb [79].

### 3.4. Improving CDC with the Introduction of Mutations

#### 3.4.1. Hexabodies to Improve CDC Induction by Stabilizing IgG Hexamers

As described in Section 2.4, C1q recruitment to induce CDC is substantially enhanced by either IgM or multimeric IgGs. Via a structure analysis, position E345 came into focus for its role in the Fc–Fc interaction needed for multimerization [38]. When the acidic glutamate is substituted with basic amino acids, such as lysine or arginine, it better complements the local electric charges of the neighboring IgG’s Fc region and, thus, forms more multimers [80]. In parallel, a substitution at position 430 from glutamate to glycine (E430G) was identified to enhance multimerization and C1q recruitment [38,81]. This engineering strategy using either E430G or the combination E430G/E435K is exploited under the name Hexabody platform [82]. GEN1029 (formerly Hexabody-DR5/DR5) harbors the E430G modification and is a mixture of two different mAbs targeting DR5. It is being tested in phase 1/phase 2 trials against several cancers [83].

#### 3.4.2. Other Mutations to Improve C1q Binding

The positions K326 and F333 were identified to be involved in C1q binding in the above-mentioned alanine scanning mutagenesis [72]. The K326W/F333S modification demonstrated increased CDC but was accompanied by lower ADCC [84,85]. However, when mutating both positions to alanine, ADCC induction was not impacted [58]. Although variants also harboring S267E, H268F or S324T were identified to improve CDC, clinical development was not initiated for any of these Fc-engineered formats [58].

### 3.5. Modulating Feedback Inhibition

As the only inhibitory ITIM-linked FcγR, FcγRIIB is undoubtedly an important modulator within the immune system. The whole plethora of activities and functions in which FcγRIIB is involved are still under investigation, but, in general, two therapeutical principles have been developed: reducing the binding of IgGs to FcγRIIB to prevent the inhibitory effects of therapeutic mAbs targeting tumor cells and the direct targeting of FcγRIIB to achieve the inhibition of specific IG-producing B cells in allergies. Recently, a third principle evolved: increasing FcγRIIB affinity for agonistic immune checkpoint therapy.

#### 3.5.1. Reducing FcγRIIB Affinity to Prevent Inhibitory Effects in Tumor Therapy

The reduced efficacy of tumor-targeting mAbs was associated with the expression of FcγRIIB [43]. Thus, Margetuximab (Margenza) was developed with the mutations L235V/F243L/R292P/Y300L/P396L introduced into the chimeric IgG1 backbone to reduce FcγRIIB interaction and, in parallel, to increase FcγRIIIA activity [86]. Margetuximab targets HER2 in several HER2-positive cancers, and it received orphan drug designation in 2020. Subsequent studies revealed that Margetuximab reduces inhibitory effector T cells and that FcγRIIIA interactions are more universal regarding ethnic polymorphism than non-engineered backbones [87].

#### 3.5.2. Increasing FcγRIIB Affinity for Agonistic Tumor Immune Therapy

Although Margetuximab is an example of a tumor-targeting mAb with reduced FcγRIIB interaction, it should be noted that there is growing evidence that the efficacy of immune checkpoint agonistic mAbs benefits from FcγRIIB crosslinking [44,88]. While blocking inhibitory immune checkpoints like PD-L1 or CTLA-4 is successful in clinical use, the clinical development of agonistic Abs against co-stimulatory pathways is lagging behind [89]. Hence, several approaches are ongoing to improve the affinity to FcγRIIB for targets such as CD137/4-1BB. LVGN6051 is an anti-CD137 IgG4 with undisclosed mutations for improved FcγRIIB crosslinking. It is currently under clinical investigation for the therapy of solid tumors [90,91,92]. In preclinical studies with LVGN6051, the advantage of FcγRIIB crosslinking was well demonstrated, and mAb-activated T cells well eliminated the cancer [89].

#### 3.5.3. Increasing FcγRIIB Affinity for the Therapy of Allergic Diseases

While inhibition through FcγRIIB may be another breakthrough in immune checkpoint cancer therapy, FcγRIIB inhibition has the potential to be the next-generation therapy in allergies. In an extensive screening study, the combination of the mutations S267E/L328F (SELF) improved the binding of IgG1 to FcγRIIB by 430-fold [93]. Relevant for potential use in tumor therapy is that SELF-modified IgG1 mAbs also demonstrated a significant lower binding to one FcγRIIIA polymorphism variant and only minor alterations to FcγRI and FcγRIIA [93]. AIMab7195 (formerly Xmab7195), an affinity maturated version of Omalizumab, is an anti-IgE humanized IgG1 harboring the S267E/L328F mutations [94]. Omalizumab is a clinically well-established anti-IgE antibody. By targeting IgE, AIMab7195 crosslinks the membrane-bound IgE with FcγRIIB on IgE-producing plasma cells to inhibit further IgE production [94]. A secondary effect of AIMab7195 is the neutralization of free IgE by blocking IgE from interacting with its receptor on immune cells. Although AIMab7195 is still under phase 1 investigation for allergic asthma and atopic diseases, it was recently licensed exclusively for the development of next-generation food allergy treatments [95].

### 3.6. Reducing and Disabling the Fc-Mediated Effector Functions

As already pointed out above, the clinical experience with targeting immune checkpoints completely changed the view that mAb-based tumor therapy must be associated with as many ITAM- or C1q-linked effector functions as possible. By contrast, blocking the checkpoint molecules in combination with reduced activatory effector functions and, if applicable, with increased inhibitory effector functions was demonstrated to reactivate tumor-reactive immune cells [44]. Thus, many mAbs targeting PD-1, e.g., Pembrolizumab or Nivolumab, are IgG4s exhibiting less activating effector functions and a loss of CDC induction. In addition, aglycosylated mAbs such as anti-PD-L1 Atezolizumab have significantly reduced effector functions. It should be noted that aglycosylated IgGs still bind FcγRI and that the effector functions of IgG4 are reduced but not eliminated. Clearly, the use of fragments or domain antibodies devoid of an Fc region may abolish all Fc-related effector functions, but this is challenging regarding the appropriate elimination half-life needed for efficacy. Hence, Fc engineering could shape antibodies disabled for all Fc-mediated effector functions but with a long half-life. In an earlier study, L234 and L235 were identified as import residues for interaction with all FcγRs [96]. Replacing the leucine at these two positions with alanine (L234A/L235A; LALA) resulted in a significant loss of binding to all low-affinity FcγRs, as well as in a significant reduction in binding to C1q and the high-affinity FcγRI [96]. The therapeutical antibodies Spesolimab (Spevigo) and Teplizumab (Tzield) have a LALA-modified IgG1 Fc region. Spesolimab targets the IL36 receptor (IL36R) to block IL36-mediated signaling in psoriasis, for which it was approved in 2022. The elimination of CD36R-positive receptors is not the aim since this receptor is also expressed on the surface of keratinocytes, the respiratory epithelium, neuronal tissue and monocytes [97]. Teplizumab was approved in 2022 to delay the development of autoimmune type 1 diabetes in young children up to two years old. The mechanism of action here is to block CD3 on T cells as a rather mild form of immune suppression. Agonistic effects inducing T-cell apoptosis may also contribute to its activity [98].

Site P329 also contributes to FcγR interaction and the mutation P329G also called PG [99]. Expressed as a variant combined with LALA as LALAPG (L234A/L235A/P329G), ADCC was fully eliminated. But not only substitutions with alanine at positions 234 and 235 result in abrogated effector function. L234F/L235E in combination with the point mutation P331S, which is close to the PG site, results in an Fc-engineered antibody with a loss of binding to all FcγRs. This L234F/L235E/P331S variant is found in the therapeutical human IgG1 Durvalumab (Imfinzi) targeting the immune checkpoint PD-L1 [100,101]. Durvalumab was approved in 2017 for tumor therapy.

Last but not least, substitution with valine at position 234, in combination with L235A, supplemented with E233P and S267K and the deletion of G236, is found in the IgG1 backbone of the XmAb platform [44]. Interestingly the combination E233P/L234V/L235A was taken from a human IgG2. Eight XmAb candidates with this reduced effector function format are in clinical phase 1 or 2 trials, and two more are in preclinical stages [102].

## 4. Fc Engineering to Alter Half-Life

### 4.1. Modified Fc–FcRn Interactions and Their Clinical Implications

As described in Section 2.6, IgGs have a longer half-life than other serum proteins, and it can extend up to 3 weeks due to their interaction with FcRn protecting them from degradation [103]. The interaction between FcRn and Fc takes place at a very narrow range of pH, that is, within the range of 5.5–6.0 [104]. Thus, an increase in this interaction will result in a prolonged serum half-life and in increased transport to mucus. Such half-life-extended IgGs can protect susceptible or immunocompromised patients for a longer period against pathogens such as viruses [105].

However, inhibiting the interaction of IgGs to FcRn is also a relevant therapeutical principle to wash out autoreactive IgGs in autoimmune diseases such as *myasthenia gravis*.

### 4.2. Half-Life-Extended Therapeutic Antibodies

Countless studies have been performed to analyze distinct Fc-based mutations and their effects on FcRn binding and half-life. Among those, the mutations at positions M252Y, S254T and T256E in human IgG1 achieved a 10-fold lower K_D_ and a 3-fold reduced apparent equilibrium rate constant [58]. This combination of mutations is referred to as YTE [106]. In cynomolgus monkeys, YTE-mutated IgG1 demonstrated a 4-fold longer serum half-life [107], and, in a phase 1 double-blind study, a comparable 4–5-fold increased serum half-life of 80–120 days was demonstrated [108]. The investigated IgG of this latter study was directed against *Staphylococcus aureus* alpha-toxin. When the YTE mutations were introduced to the mAb Motavizumab targeting the respiratory syncytial virus (RSV), the half-life was extended in humans by 2–4-fold [109]. It is important to note that YTE-modified mAbs are reported to display significantly lower ADCC activity [107], hence binding to FcγRIIIA.

YTE-modified Nirsevimab (Beyfortus) was approved in 2022 and was demonstrated in several clinical studies to efficiently protect infants and toddlers from RSV infections and associated complications because of its extended half-life [110]. The antibodies Tixagevimab and Cilgavimab are both half-life-extended via YTE mutations, and both bind to distinct epitopes on the SARS-CoV-2 spike protein originally derived from convalescent patients after SARS-CoV-2 infection [100]. Further, the Fc regions of both antibodies are reported to have reduced FcγR and C1q binding. Evusheld is a medical product containing a combination of these two antibodies, and it was approved in 2022. Evusheld demonstrated a statistically significant reduction in the risk of developing symptomatic COVID-19 in a phase 3 pre-exposure prevention trial with over 5000 enrolled participants [100,111]. Livilimab is a YTE-modified anti-interleukin-6 mAb for the therapy of rheumatoid arthritis, but it has also demonstrated activity against the immune-related complications of severe SARS-CoV-2 infection. It was approved in Russia in 2020 for COVID-19 [112]. Netakimab is a humanized YTE-modified anti-IL-17 IgG1/κ type, in which the variable domain VH is substituted with a variable VHH domain of a single-domain antibody [106,113]. Netakimab was evaluated in a phase 3 clinical trial in patients with moderate-to-severe vulgar psoriasis and demonstrated high efficiency and a high safety profile [114]. This half-life-extended mAb is already approved in Russia for psoriasis and has a pending approval for ankylosing spondylitis.

A second set of Fc-based mutations that decrease the K_D_ of the IgG1-FcRn complex by 11-fold is M428L/N434S in human IgG1, also called LS mutations or Xtend^TM^ [115,116]. In cynomolgus monkeys, a 3-fold increase in half-life was observed, but mucosal mAb concentrations were also increased in non-human primates [117]. Compared with YTE, LS mutations do not significantly alter ADCC activity [58].

Ravulizumab (Ultomiris) in an LS-modified anti-C5 IgG2/4κ that was approved in 2018 for the treatment of paroxysmal nocturnal hemoglobinuria [118]. The reported serum half-life was bout 50 days. LS-modified Sotrovimab (Xevudy) is a human IgG1κ neutralizing SARS-CoV-2′s spike protein [119]. Sotrovimab was approved in 2021 under an FDA emergency use authorization, but with the occurrence of the Omicron variant of SARS-CoV-2, the FDA canceled the authorization in 2022 due to a lack of efficacy. Amubarvimab and Romlusevimab are two neutralizing human IgG1 monoclonal antibodies targeting SARS-CoV-2, with both containing the YTE modification, and they were approved as a combination product in China in 2021 [120]. In clinical studies, Amubarvimab revealed a somewhat shorter mean terminal half-life of 44.6–48.6 days, while Romlusevimab achieved 72.2–83.0 days. Finally, several broadly neutralizing antibodies (bNAbs) against human immunodeficiency virus (HIV) are half-life-extended via LS modification and under clinical investigation in different clinical trials [121].

It should be noted that the mutations M428L/N434A (so-called LA) also increase the serum half-life of IgGs. Adintrevimab is one of such LA-modified mAbs and is directed against SARS-CoV-2, but it is still under clinical development [106,122,123].

### 4.3. Antibodies That Block FcRn Binding to Wash out Autoreactive Endogenous IgGs

Efgartigimod alfa (Vyvgart) is a human recombinant IgG1-derived Fc fragment produced in CHO cells, and it harbors five mutations at positions M252Y, S254T, T256E, H433K and N434F that significantly increase the binding of this engineered Fc domain to FcRn [124,125]. In fact, this interaction is so tight that, even at low pH, Efgartigimod is not released any more from FcRn; hence, the receptor is blocked for the recycling of endogenous IgGs. Therefore, the amount of all endogenous-circulating IgG subclasses decreases, and autoantibodies such as those responsible for Myasthenia gravis are washed out. In 2021, Efgartigimod alfa was approved as first-in-class medication for the treatment of Myasthenia gravis. Interestingly, in a clinical trial, it was reported that only IgG concentrations were reduced by Efgartigimod, without affecting the levels of serum albumin [126].

Before Efgartigimod was developed and approved, Myasthenia gravis was treated with high doses of IVIg to saturate all FcRn molecules with high concentrations of IgGs thereby limiting the recycling of all IgGs and, thus, increasing IgG clearance [45].

While Efgartigimod blocks FcRn with an Fc-engineered region, other mAbs were clinically developed for *myasthenia gravis* therapy, which bind pH-independently FcRn with their paratop. These antibodies include Rozanolixizumab, an IgG4 approved in 2023; Batoclimab, a fully human IgG1 with an engineered Fc region to reduce ADCC, which is in a phase 3 trial and under review in China; and Nipocalimab, an aglycosylated fully human IgG1 in a phase 2 trial [127,128,129,130,131]. The humanized anti-FcRn IgG4κ Orilanolimab (formerly SYNT001) is Fc-engineered with the S241P mutation to reduce FcγR-mediated effector functions [132]. In preclinical studies, Orilanolimab was well tolerated and proved effective in non-human primates [132]. It was clinically developed as therapy for pemphigus, a group of rare blistering autoimmune diseases that are triggered by autoreactive antibodies. Phase 2 studies in patients with pemphigus foliaceus and pemphigus vulgaris demonstrated that SYNT001 reduced antibody levels and disease activity [133]. However, Orilanolimab was withdrawn.

In contrast to the smaller Fc-only Efgartigimod, all these full IgGs appear to more strongly affect albumin recycling. Rozanolixizumab slightly decreases albumin levels, however not significantly [125]. Batoclimab and Nipocalimab both decrease albumin but in a reversible manner and are mostly asymptomatic [131,134]. This is likely because there are different binding sites for IgG and albumin in the FcRn molecule. As an isolated Fc domain, Efgartigimod is rather small, and sterical hindrance with albumin binding is therefore possible but not too likely to occur.

## 5. Fc Engineering to Achieve Bispecific Heterodimeric IgG-Based Antibodies

Bispecific antibodies are engineered to bind to two different antigens with two different antigen-binding sites, a principle that is, apart from IgG4 Fab arm exchange, not found in endogenous mammalian immunoglobulins [135,136]. The ability to bind to two different targets allows bispecific antibodies to bring two structures in close spatial vicinity so that they can interact with each other. These two structures can be two proteins or even two cells. In particular, targeting a T cell with an agonistic paratop in parallel to targeting a tumor cell is a successful therapeutic principle [135,137]. Such bispecific therapeutics recruiting and activating T cells to attack tumor cells are denominated bispecific T-cell engagers (BiTEs), a similar principle to chimeric antigen receptor (CAR) T cells [138]. It should be noted that some authors use the term BiTE to exclusively describe diabodies (bispecific recombinant antibodies expressed as one single chain and devoid of the Fc region). Moreover, the bispecific concept can also be used for drug targeting, such as transcytosis via the blood–brain barrier, with one paratop binding the transporter and the other the pharmacological target [139]. A very interesting clinical development in this field is Trontinemab (formerly RO7126209), an updated bispecific version of the anti-amyloid mAb Gantenerumab, engineered to cross the blood–brain barrier using “brain shuttle” technology [140]. Another use of the bispecific concept is in connection with immunoliposomes. Bispecific immunoliposomes have shown increased cell interaction and cytotoxic drug delivery compared to monospecific immunoliposomes [141].

The paratope of any endogenous antibody is created by somatic VD(J) recombination and is produced exclusively by one B-cell clone. The challenge of bispecific antibodies is to introduce two different VHs and VLs into identical CH1-CH2-CH3 and CL, respectively, to be expressed correctly in a distinct symmetry [137]. Combinatorial considerations reveal that “just” expressing two different heavy chains and two different light chains results in 16 different combinations equivalent to 10 different molecules. Only 1 out of these 10 molecules is the desired bispecific product. Thus, the co-expression of identical chains, which differ only in their variable fragment (Fv), is not an optimal choice. Nevertheless, Catumaxomab (Removab) was the first BiTE in clinical use targeting EPCAM and CD3. Catumaxab was the product of two fused hybridoma cells, one from a rat and one from a mouse, a so-called quadroma cell. It was approved for malignant ascites in 2009, but it is no longer in use [23].

Despite the fact that a plethora of different formats and building blocks are described (see [137] for a review), we focus here on the formats of IgG-based asymmetric heterodimeric bispecific antibodies, which result from smart Fc engineering. While symmetric IgG-based bispecific antibodies fuse the second Fv by different means to an IgG, a heteromeric design of the heavy chains is needed for asymmetric bispecific antibodies. A design solution to achieve heterodimeric heavy chains is to use either steric or electrostatic effects or a combination thereof [137].

### 5.1. Bispecific Antibody Designs for a Heterodimeric Assembly during Expression

**Figure 3:**
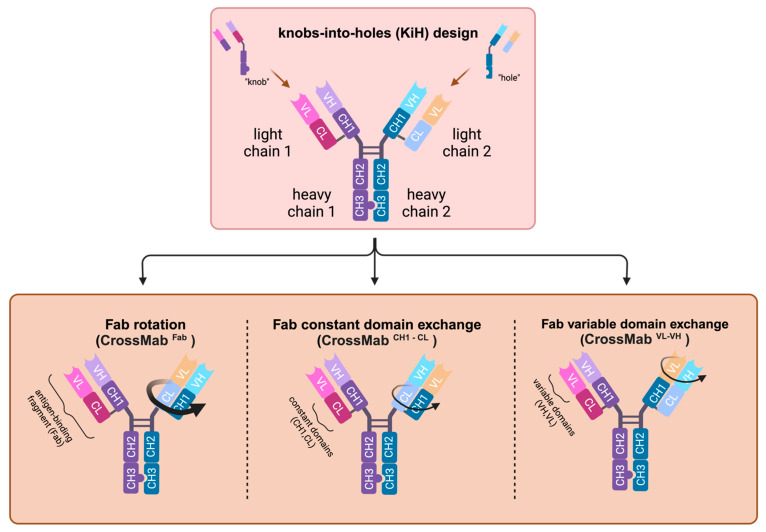
”Knob-into-holes” and CrossMab design of heterodimeric bispecific antibodies. “Knobs” (located on heavy chain 1) and “holes” (found on heavy chain 2) are created using bulky and small amino acids, respectively, in the CH3 region. This design strategy enhances the likelihood of a heterodimeric assembly. The CrossMab technology is employed to fix a light chain to its respective heavy chain. This involves rotating the entire Fab structure, resulting in a “heavy chain” composed of CH3-CH2-CL-VL and a “light chain” consisting of CH1-CL. Since CH1-CL cannot assemble with CH1-VH on the other heavy chain, the “light chain” is affixed to the rotated heavy chain (CrossMab^ab^). Similarly, this can also be achieved by exchanging/rotating only one domain of the light chain, either the CL (CrossMab^CH1-CL^) or the VL (CrossMab^VL-VH^). Created with BioRender.com.

The concept of steric hindrance was used for the “knobs-into-holes” (KiH) design by replacing a small amino acid in one heavy chain with a large one and vice versa on the opposite location in the other heavy chain, thus creating a knob and a hole [142]. The substitution of T366Y in one chain and Y407T in the other chain worked best, but still quite a few undesired monospecifics occurred. Therefore, further mutations were introduced to increase the hole, and Y366 was replaced with W to have a bulkier knob stabilizing the heterodimerization. The hole-bearing heavy chain containing Y349C, T366S, L368A and Y407V and the knob containing the S354C and T366W mutations resulted in a yield of over 95% during production [143] (Figure 3). The effector functions were predominantly retained.

For the first formats, a single-chain variable fragment (scFv) was fused to one of the heavy chains, or only one light chain was used as the so-called common light chain. The development of the “Crossmab” design, based on the above-mentioned KiH design, solved the problem where the light chain had not been fixed to the correct paratop’s heavy chain. Although this is not related to Fc engineering, here, a short outline of how this was achieved using smart molecular engineering is provided: either the whole light chain of one Fab arm is exchanged by the CH1-VH of the corresponding heavy chain, or just the VH by the VL or the CH1 by the CL is exchanged [144]. Glofitamab (Columvi) is a bispecific IgG1 BiTE developed with the CrossMab platform targeting CD20 and CD3 and approved in 2023 for the therapy of diffuse large-B-cell lymphoma [145].

Meanwhile, the KiH concept was transferred from IgG1 to IgG4 to also tune the effector functions of bispecific antibodies [146]. Of course, an effector can also be silenced by mutations: The highly Fc-engineered Faricimab (Vabysmo) is a bispecific IgG1 based on the CrossMab platform simultaneously targeting VEGF-A and Ang-2, thus being a bispecific antibody that is not a BiTE. It was approved in 2022 for the treatment of age-related macular degeneration and diabetic macular edema. The pharmacodynamic mechanism is to bind and block VEGF and angiopoietin-2 (Ang-2) at the macular region. Therefore, this bispecific mAb is applied via an intravitreal injection into the eye [147]. As the eye is a part of the central nervous system, pro-inflammatory effector functions are undesired. Thus, the Faricimab Fc region has been modified with undisclosed mutations to reduce binding to FcγR to reduce CDC, ADCC and ADCP [148]. In addition, vitreal elimination is mediated by FcRn. So, in contrast to the therapeutic antibodies located in serum, a decreased affinity to FcRn results in an extended half-life in this compartment. Therefore, the Fc engineering of Faricimab also aims to reduce FcRn binding with an estimated mean apparent systemic half-life of 7.5 days [149].

Apart from hydrophobic interactions, electrostatic charge pairing can also be engineered to strengthen the formation of heterodimers and to disfavor homodimers [137]. Rather efficient is the substitution with aspartate or glutamate in one CH3 and lysine in the other CH3 (K409D/K392D; D399K/E356K) named DDKK or (L351D/L368E; L351K/T366) DEKK [150,151]. Introducing glutamic or aspartic acid in one CH3 at positions 349, 351, 355 and 368 and lysine in the other CH3 at 349, 351, 355 and 368 results in so-called biclonics [152]. The biclonic MCLA-128 targeting HER2 and HER3, thus not a BiTE, is being evaluated in clinical trials [152]. Moreover, charge pairing may also be introduced into the hinge region and in subclasses other than IgG1 [137].

Also, EW-RVT formats are known to stabilize bispecific heterodimers [153]. Here, the endogenous hydrophobic core of CH3-CH3 interactions is retained and the surrounding residues are replaced to achieve bulky knobs and small holes, respectively, and to also achieve charge pairing (K360E/K409W; Q347R/D399V/F405T) with expression yields of bispecific antibodies of up to 90% [154]. The XmAb platform achieves heavy-chain heterodimerization by substituting S364H and F405A in one CH3 domain and Y349T and T394F in the other CH3 domain [155]. XmAb-14045 targeting CD3 and CD123 is being evaluated in clinical trials for the treatment of hematological malignancies [156].

The probably best-known non-BiTE breakthrough bispecific antibody is Emicizumab (Hemlibra), approved in 2017 for hemophilia A treatment in patients with inhibiting anti-factor VIII (FVIII) antibodies [157]. Bringing the activated coagulation factor IXa (FIXa) in close vicinity to factor X (FX) enables the FIXa-mediated proteolytic activation of FX to FXa; this is usually the function of FVIII, which is absent in hemophilia A. Emicizumab was designed by engineering a common light chain (CDR1 and CDR3 bind to FIXa, and CDR2 binds to FX) and by engineering differentially mutated heavy chains to favor heterodimers via electric repulsion and attraction, called ART-Ig (asymmetric reengineering technology-immunoglobulin) [158,159]. In addition, isometric point engineering was performed to isolate the desired bispecific IgG from the unwanted byproduct IgGs.

Last but not least, we want to mention SEEDbodies as examples of IgG-IgA crossovers [160]. The IgG-IgA chimeras designed via the strand-exchange engineered domain (SEED) are composed of alternating IgG and IgA segments but are trimmed for IgG-comparable FcRn binding [161].

### 5.2. Bispecific Antibodies with Manufacturing-Specific Steps for Heterodimerization

Fab arm exchange occurs naturally in IgG4 via a reduction in the hinge disulfide bonds, the random exchange of the half IgG4 molecules called Fab arms and reassembly into full IgG4 with the re-oxidation of the disulfide bonds [136]. The DuoBody platform uses this naturally occurring phenomenon to generate bispecific antibodies via controlled Fab arm exchange (cFAE) [137,162]. Introducing K409R to the one and F405L to the other heavy chain, monospecific antibodies are expressed, mildly reduced to Fab arms and reassembled by mixing with the respective other monospecific Fab arm. The different single mutations in each heavy chain favors the formation of heterodimers, with a yield of about 95%. Epcoritamab (Epkinly) is a DuoBody-based BiTE targeting CD20 and CD3 that was approved in 2023 for the treatment of diffuse large-B-cell lymphoma [163].

Another approach also focuses on post-production assembly rather than stabilizing heterodimers: The mutations H435R/Y436F are introduced into one of the monospecific antibodies (Fc*) needed for a bispecific antibody. This combination also occurs in IgG3 and reduces the affinity to protein A. After the reassembly of the Fab arms, the different fractions Fc-Fc, Fc*-Fc and Fc*-Fc* are separated using protein A chromatography similar to the manufacturing of rat/mouse quadroma-derived bispecific antibodies [137,164].

## 6. Conclusions and Outlook

Recent advances in the Fc engineering of modern therapeutic antibodies can be considered the next generation of antibody therapy. These molecules demonstrate superior clinical outcomes by using mutations, a combination of mutations or glycoengineering to finetune their activity and tailor it to the needs of the therapy. These next-generation antibodies will dominate clinical practice within the coming years.

The field of antibody therapy is rapidly evolving, and it is likely that we will see even more effective and targeted antibody-based therapies in the future. Apart from the here-mentioned clinical examples, there are some future trends that have a good chance of being translated to clinical products, such as IgA–IgG hybrids or chimeras, for more IgA-related effector functions, such as NETosis, complement and mucosal immunity [165]. The benefit of IgA is stronger FcR signaling, and the presence of both Fcα and Fcγ would give the resulting chimeric antibodies the ability to bind to a greater number of FcR molecules than the parental antibodies, resulting in the increased killing of target cells, such as cancer. Moreover, IgA–IgG chimeras containing an Fcα-Fcγ domain could be effective as anti-infective agents, particularly against HIV-1.

In general, the therapeutic use of more Ig classes is likely to emerge, as, during the SARS-CoV-2 pandemic, polyclonal IgM-enriched serum-derived antibody products demonstrated the therapeutic possibilities of other Ig classes; however, not all of the studies reached their endpoints with statistical significance [166,167,168].

BiTEs are about to revolutionize tumor therapy, and trispecific antibodies, with a third paratope fused as scFv either to the Fc domain or to one paratope, are currently under clinical investigation [169,170,171].

Last but not least, only the first steps have been taken in the large field of glycoengineering, and there is far more to come as soon as we better understand the endogenous regulation of immunity by different glycans [172,173].

The global monoclonal antibody market size was valued at roughly USD 147 billion in 2020 and is projected to reach USD 391 billion by 2030 [174]. An exciting part of this future is devoted to Fc-engineered antibodies.

## Data Availability

The data presented in this study are available in this article (and Supplementary Materials).

## Supplementary Materials

The following supporting information can be downloaded at: https://www.mdpi.com/article/10.3390/pharmaceutics15102402/s1, Table S1: List of clinical antibodies discussed in this review.

## Abbreviations

ADCC	antibody-dependent cellular cytotoxicity
ADCP	antibody-dependent cellular phagocytosis
Ang-2	angiopoietin-2
ART-Ig	asymmetric reengineering technology-immunoglobulin
BiTE	bispecific T-cell engager
C1q	complement component 1q
CD	cluster of differentiation
CDC	complement-dependent cytotoxicity
CDR	complementarity-determining region
CH	constant region of heavy chain
CHO	Chinese hamster ovarian
CTLA-4	cytotoxic T-lymphocyte-associated protein-4
Fab	fragment antigen binding
Fc	fragment crystallizable
Fcγ	fragment crystallizable gamma
FcRn	neonatal Fc receptor
FcγR	Fc gamma receptor
FIXa	activated coagulation factor IXa
FUT	fucosyltransferase
FVIII	coagulation factor VIII
FX	coagulation factor X
FXa	activated coagulation factor X
GlcNAc	N-acetylglucosamine
GnTIII	N-acetylglucosaminyltransferase III
HAMA	human anti-mouse antibody
HER2	human epidermal growth factor receptor-2
HIV	human immunodeficiency virus
IgG	immunoglobulin G
IL	interleukin
ITAM	tyrosine-based activation motif
ITIM	immunoreceptor tyrosine-based inhibitory motif
IVIg	intravenous immunoglobulin G
κ	kappa light chain
kDa	kilodalton (a unit of molecular weight)
KiH	“knob-into-holes” design
mAbs	monoclonal antibodies
MAC	membrane attack complex
MHC	major histocompatibility complex 4
NET	neutrophil extracellular trap
NK cells	natural killer cells
PD-1	programmed cell death protein-1
PD-L1	programmed cell death ligand 1
scFv	single-chain variable fragment
sdAb	single-domain antibody
SEED	strand-exchange engineered domain
TNFα	tumor necrosis factor-alpha
VEGF	vascular endothelial growth factor
VH	variable region of heavy chain
